# Assessing Unmet Needs of Patients With Chronic Pain: The Development and Validation of the Needs Evaluation Questionnaire for Chronic Pain

**DOI:** 10.1155/prm/6906748

**Published:** 2026-07-29

**Authors:** Francesca Chiesi, Costanza Gori, Caterina Primi, Carlotta Tagliaferro, Michael Tenti, Andrea Bonacchi

**Affiliations:** ^1^ Department of Neuroscience, Psychology, Drug, and Child’s Health (NEUROFARBA), University of Florence, Florence, Italy, unifi.it; ^2^ Institute for Research on Pain, ISAL Foundation, Rimini, Italy; ^3^ Centro Studi e Ricerca Synthesis, Associazione Promozione Sociale Sul Sentiero, Florence, Italy

## Abstract

**Background:**

For its high prevalence, diagnostic and therapeutic challenges, and serious burden on affected individuals and societies, chronic pain is considered a public health priority worldwide. However, there is a paucity of data regarding unmet needs of chronic pain sufferers. The aim of the present study was to develop and validate the Needs Evaluation Questionnaire for Chronic Pain (NEQ‐CP) for assessing unmet needs of chronic pain patients.

**Methods:**

The questionnaire development and validation followed some steps: (i) operational definition and item formulation; (ii) investigation about item relevance and comprehensibility; (iii) item selection based on comprehensibility and content validity; (iv) item selection based on item descriptives and item response theory; (v) reliability and validity testing. Participants (an online sample of 521 Italian patients with chronic pain associated with various diseases) were cross‐sectionally administered the initial version of the questionnaire and some scales chosen for validity testing.

**Results:**

Content validity testing led to the identification of a provisional pool of items. Item analysis and item response theory analyses allowed the selection of 21 items for the final version of the NEQ‐CP. The questionnaire showed good general and local reliability, and construct and criterion validity evidence were provided.

**Conclusions:**

The developed and validated questionnaire is an effective and easy‐to‐use tool in the assessment of the unmet needs of patients with chronic pain. It can be used to identify individuals requiring immediate support and to assist those involved in health policies and research in allocating resources to areas with greater and more urgent needs.

## 1. Introduction

Chronic pain (CP), defined as pain persisting or recurring for longer than 3 months [1], is a complex condition [2] affecting a significant proportion of the adult population, approximately 20% in Western countries [3] and 24.1% in Italy [4]. CP can severely impact health and quality of life, leading to functional impairment, sleep disturbances, depression, anxiety, social isolation, and job loss [5]. Despite increasing clinical awareness, CP remains challenging to diagnose and treat, with many patients experiencing delayed diagnoses, partially effective treatments, and side effects that further reduce quality of life [4, 6, 7]. Moreover, patients often face stigmatization and disbelief from clinicians, family members, and coworkers, exacerbating psychological distress and social isolation [8, 9].

Given these challenges, patients with CP frequently experience substantial unmet needs, defined as the lack of services or support that a patient feels necessary to achieve an adequate quality of life [10]. According to Osse et al. [11], an unmet need reflects a patient’s desire for support concerning perceived problems, often related to information and communication with healthcare providers [12], assistance/care from healthcare systems [13], and psychosocial support [12]. Unfortunately, few studies have investigated these needs in the field of CP and they focus solely on the doctors’ perspective [14], unmet needs regarding medical treatment [15] or pain management [16], or specific diseases [17, 18]. Moreover, they often use qualitative approaches, nonvalidated scales, and questionnaires (e.g., [14, 17, 18]). Only a qualitative–quantitative study conducted on women with autoimmune rheumatic disorders used a validated instrument, but this questionnaire focused solely on informational needs [19]. Nonetheless, the study found a high number of unmet informational needs among participants, particularly related to pain, mobility, and the impact of the disease on them and their children during pregnancy and early parenting.

Of note, evidence from other fields has shown that unmet needs are associated with lower patient–provider communication self‐efficacy and reduced satisfaction with care, while a decrease in unmet needs contributes to improved motivation for treatment [20, 21].

Evaluating unmet needs in CP could therefore be crucial to enhance quality of life, treatment outcomes, and inform healthcare policy and resource allocation. While several patient‐reported outcome measures in CP address domains such as motivation for pain relief, patient involvement in care, trust in physicians, and perceived treatment helpfulness, they do not specifically assess patients’ unmet needs [22, 23]. To the best of our knowledge, no validated questionnaire currently comprehensively captures the broad spectrum of unmet needs experienced by CP patients. To address this gap, we propose adapting the *Needs Evaluation Questionnaire for Chronic Pain* (NEQ‐CP) from the original *Needs Evaluation Questionnaire* (NEQ [24, 25]), designed to assess cancer patients’ needs. The NEQ is a brief, self‐administered questionnaire widely used in oncology settings to evaluate patients’ informative, communicative, relational, material, psychological, and care‐related needs. The original 23‐item NEQ was expanded to 27 items for outpatient cancer populations and has demonstrated good psychometric properties [24, 26, 27]. Given the shared challenges across CP and cancer populations—such as emotional burden, communication difficulties, and care coordination—the NEQ provides a sound foundation for adaptation to the CP population. However, specific aspects of the CP experience (e.g., invisibility of symptoms, stigma, lack of hospitalizations, fragmented care pathways) necessitate targeted modifications to ensure relevance and sensitivity.

The aim of the present study is therefore to adapt and validate the NEQ for use in CP populations, providing a validated instrument to identify unmet needs, guide clinical practice, and inform service planning.

## 2. Methods

This cross‐sectional study was conducted in accordance with the guidelines of the Declaration of Helsinki and its subsequent amendments. Ethics approval was obtained from the Ethics Committee of the University of Florence (n. 233, December 1, 2022).

The NEQ‐CP was developed and validated considering the guidelines for scale development and testing proposed by Tsang et al. [28]. The study adheres to the reporting standards of Streiner and Kottner [29] and was evaluated in line with relevant COSMIN recommendations for patient‐reported outcome measures, specifically with regard to content validity, structural validity, internal consistency, and construct validity. The development of the NEQ‐CP followed two main phases: (i) development of a preliminary version of the NEQ‐CP, which involved item development, the investigation of their relevance and comprehensibility, and item selection based on comprehensibility and content validity; (ii) validation of the NEQ‐CP, which included item selection based on item descriptives and item response theory (IRT), and reliability and validity testing.

### 2.1. Development of a Preliminary Version of the NEQ‐CP

The NEQ‐CP questionnaire was developed starting from the NEQ adapted for outpatients with cancer by Bonacchi et al. [27]. All 27 items were initially included in the NEQ‐CP. In the preliminary phase assessing item comprehensibility and relevance, a Likert‐type response format was used; in the subsequent validation study, the response scale was dichotomous (yes/no), in line with the original format proposed by Tamburini et al. [24].

The 27 items of the original NEQ were administered to a sample (Sample A, *n* = 59) of patients and CP specialists to investigate the relevance of the items in the context of CP and to assess item comprehensibility. The patients’ group consisted of people suffering from CP (*n* = 35), recruited from the CP patients’ association affiliated with the ISAL Foundation (Institute of Algological Sciences); an Italian nonprofit organization dedicated to research, education, and patient support in the field of CP. Through its national network of patient associations and outreach activities, the Foundation interacts with individuals living with CP who are followed in different healthcare settings. Consequently, the recruited participants represented a heterogeneous group of patients with experience across different levels of care, including both primary care management and specialized pain services. The sample of CP specialists (*n* = 24) included pain physicians, psychologists, nurses, and other specialists among the scientific board and teachers at the ISAL School in Algological Sciences. Patients and specialists were asked to rate the relevance (indicator of content validity) and comprehensibility of each item when referred to CP. Ratings were on a 4‐point Likert scale (1 = very irrelevant/very unclear; 2 = irrelevant/unclear; 3 = relevant/clear; 4 = very relevant/very clear). Additional open‐ended questions regarding suggestions for potential changes to existing items and relevant patients’ needs in the context of CP not already assessed were included at the end of the questionnaire.

Two of the authors of the present study (Michael Tenti and Andrea Bonacchi) independently reviewed the feedback provided by participants in Sample A in order to identify potential adjustments to the wording of the original items. They also analyzed the needs considered relevant by participants but not yet addressed in the original NEQ and reformulated them into new items capable of capturing these specific aspects. Any discrepancies in their evaluations were resolved through discussion and comparison with a third author (Francesca Chiesi). This process led to the development of a preliminary version of the NEQ‐CP, which included the original 27 NEQ items and the newly proposed items. This preliminary version was then administered to a different sample (Sample B, *n* = 25) composed of 11 CP specialists and 14 patients, who were asked to rate the relevance and comprehensibility of each item.

Item scores of 1 and 2 were recoded to 0, indicating “not present.” Similarly, scores of 3 and 4 were recoded to 1, indicating “present.” For each item, indices were calculated for comprehensibility (I‐CI) and content validity (I‐CVI). The I‐CI was computed as the proportion of respondents who rated the item as comprehensible, while the I‐CVI was computed as the proportion of respondents who rated the item as relevant. According to criteria proposed by Polit et al. [30], item indices (I‐CI and I‐CVI) lower than 0.78 were considered inadequate and required further analysis. Values between 0.80 and 0.89 were considered acceptable, whereas values ≥ 0.90 were considered excellent. These three phases led to identifying the provisional items of the NEQ‐CP upon which we defined the adequate sample size to undertake the next phases of the development of the NEQ‐CP.

### 2.2. Validation of the NEQ‐CP

#### 2.2.1. Participants

To ensure an adequate sample size, we considered a number of participants equal to 10 times the number of items of the NEQ‐CP. Based on this criterion, we estimated that a sample of 250–350 cases (10 cases per item, accounting for potential exclusions and additions to the original NEQ version, which may range from 25 to 35 items) would be appropriate [31, 32]. Following this estimation, the NEQ‐CP validation was conducted using a convenience online sample of 521 Italian patients with CP, recruited between February and December 2023 through websites and social pages of ISAL Foundation and other affiliated CP patients’ associations. To be eligible, participants should be ≥ 18 years old and declared to be affected by chronic noncancer pain for at least three months.

#### 2.2.2. Measures and Procedure

Before initiating the study, all participants were required to read and sign an informed consent form. Subsequently, they completed an electronic questionnaire, which was administered anonymously.

Administration time ranged from 25 to 40 min, and the online questionnaire consisted of the preliminary version of the NEQ‐CP and the following scales.

##### 2.2.2.1. Brief Five‐Item Chronic Pain Questionnaire (CP‐QUEST)

It was used to assess CP. This validated, self‐administered questionnaire has been designed to identify individuals with persistent pain lasting longer than 3 months and to gather information about pain characteristics within the general population. The first question of the CP‐QUEST screens for individuals affected by CP, asking if they have experienced persistent physical pain for over 3 months. For those who screen positive for CP, the questionnaire then asks four additional questions on: (1) pain intensity, with a 5‐level verbal scale; (2) potential triggers of CP; (3) frequency of treatments, including medications and other therapies; (4) treatment perceived effectiveness. The CP‐QUEST has demonstrated good understandability, reliability, and validity [33].

##### 2.2.2.2. Chronic Pain Grade Questionnaire–Disability (CPG‐Disability) Subscale

The “Disability” subscale of the Italian‐validated CPG [34] was used to assess the impact of CP on daily functioning. The CPG is a self‐report measure designed to evaluate the intensity of CP and its associated disability. For this study, only the CPG‐Disability subscale was employed, as pain intensity was independently assessed using the CP‐QUEST. The CPG‐Disability subscale evaluates the impact of CP on daily functioning through four items that assess the influence of pain on daily, social, and work activities. The CPG has been shown to be a valid and reliable instrument, as demonstrated in its validation study [34].

##### 2.2.2.3. Short Form‐12 Items Health Survey (SF‐12 [35])

It is an abbreviated version of the Short Form‐36 items Health Survey (SF‐36 [36]), and it evaluates perceived physical and mental health. A physical and mental score can be computed (PCS‐12 and MCS‐12, respectively), and higher scores represent greater levels of perceived health.

##### 2.2.2.4. Well‐Being Numerical Rating Scale (WB‐NRS [37])

The WB‐NRS is a brief measure of multidimensional well‐being. Respondents are asked to rate their physical, psychological, relational, spiritual, and general well‐being using a ten‐point scale (from 1 = “complete distress” to 10 = “complete well‐being”). The measure shows good psychometric properties [37, 38], and it has been translated and validated in Chinese [39], British and American English, French, German, Hungarian, Swedish, and Spanish [40].

##### 2.2.2.5. Depression Anxiety Stress Scale (DASS‐21 [41]; Italian Version [42])

It consists of three 7‐item subscales. Items are on a four‐point rating scale (0 = “does not apply to me at all,” 3 = “applies to me a lot or most of the time”). Higher scores indicate higher levels of depression, anxiety, or stress.

In the online questionnaire, participants were also asked to provide demographic and clinical information, including age, sex, education level, occupation, housing situation, area of residence, and self‐reported medical diagnosis of CP. The diagnosis could be selected from a predefined list (single‐choice format); in cases of multiple diagnoses, participants had the option to select “Multiple comorbid chronic pain conditions.”

### 2.3. Statistical Analysis

Analyses described below were conducted using SPSS Version 29.0, JASP for Windows software (Version 0.18.1), [43] and IRTPRO 6.0 [44].

#### 2.3.1. Descriptive Item Analysis

Prior to conducting the analyses, the missing values in the data were examined. For each item, the percentage of missing answers was calculated to check that they did not exceed 10% of the total answers. Listwise deletion was performed when there were > 10% of missing answers; otherwise, the arithmetic means of each item replaced the missing data [45]. Then, we calculated the item percentages of positive responses to check the variability of the responses to the items, i.e., their ability to be sufficiently informative. A very low or very high percentage indicates that respondents almost all answer the same way, so the question is not informative. In any case, since the questionnaire also aims to capture the very prevalent needs of patients with CP, we set the cutoff for high percentages at ≤ 95%. The cutoff for low percentages was ≥ 20%.

#### 2.3.2. IRT Analysis

Before performing IRT analyses, unidimensionality and local dependence assumptions were tested. The assumption of an adequate single latent factor makes it possible to estimate the latent trait. It is satisfactory for IRT parameter calibration if it explains > 20% of the variance in items [46, 47]. Thus, an explorative factor analysis (EFA) was conducted using the polychoric correlation matrix and the minimal residual (MINRES) extraction method [48], fixing the number of factors to one. The local dependency (LD) was assessed using the diagnostic *χ* ^2^ LD statistic [49]. As a rule of thumb, values of 10 or greater indicate the presence of LD. Then, the two‐parameter logistic (2PL) IRT model was applied to the data. This model was selected because it allows the estimation of both item discrimination (*a*) and item location (*b*) parameters and is appropriate for dichotomous items. The item fit under the 2PL model was tested by computing the S‐*χ* ^2^ statistics, and a level of significance equal to *α*/*n* (number of estimated parameters) was used to adjust for multiple comparisons. Under the 2PL, the location (*b*) and the discrimination (*a*) characteristics of the items were estimated by employing the marginal maximum likelihood estimation method with the expectation–maximization algorithm [50] implemented in IRTPRO. The *b* parameter can be interpreted as the “relevance” of the need described by the item and, specifically, higher values identify minor needs, while lower values represent major needs. The *a* parameter represents the discrimination ability of the item. The higher *a* is, the better the item’s ability to differentiate between people with different levels of unmet needs. Following Baker and Kim, *a* value below 0.24 is considered very low, 0.25 to 0.64 are considered low, 0.65 to 1.34 are considered moderate, 1.35 to 1.69 are considered high, and more than 1.7 are very high [51].

#### 2.3.3. Reliability

Scale classical reliability was assessed using the K‐R20 coefficient with a relative 95% confidence interval. Values below 0.70 are considered insufficient (0.70 ≤ *α* 0.79 fair; 0.80 ≤ *α* ≤ 0.89 good; *α* ≥ 0.90 excellent). Additionally, inside the IRT framework, the test information function (TIF) evaluates the precision of the test at different levels of the measured trait [52]. The associated reliability is 1 minus the inverse of the information the test provides [*r* = 1 − (*1*/*I*)]. The information curve shows graphically the local reliability of a test. To better interpret the local reliability of the questionnaire, summed scores, which represent the number of unmet needs endorsed by the respondent, were translated into IRT trait scores. These scores are on the same continuum (and metric) of the item location parameters and are obtained by a score conversion table provided by IRTPRO applying the expected a posteriori (EAP) method [53].

#### 2.3.4. Validity

Construct and criterion validity were studied through correlation analyses, calculating a total score of the NEQ‐CP. Bivariate correlations between the NEQ‐CP and subjective perception of mental and physical health, depression, stress, anxiety, and well‐being scores were computed. The *t*‐test was used for comparing therapy (yes/no) groups. Cohen’s *d* values from 0.2 to 0.5 are indicators of a small effect, values from 0.5 to 0.8 represent a medium effect, and values from 0.8 represent a large effect. Analysis of variance (ANOVA) was performed to assess the effect of therapy efficacy (yes, partial, no). The partial eta squared (ηp^2^) was used for the effect size (values lower than 0.06 suggest a small effect, values from 0.06 to 0.14 a medium effect, values from 0.14 a large effect). Both the grouping variables are deemed relevant in explaining the general level of unmet needs.

## 3. Results

### 3.1. Development of a Preliminary Version of the NEQ‐CP

The group of expert patients in Sample A (*n* = 35) consisted exclusively of women aged between 25 and 68 years (mean = 46.86, SD = 12.67), most of whom were married (57.1%), employed (63.1%), and held either a high school diploma (37.1%) or a university degree (40%). The majority of them (65.7%) reported experiencing pain for more than 10 years. The group of CP specialists in Sample A (*n* = 24) was gender‐balanced (50% female, 50% male), with ages ranging from 32 to 72 years (mean = 49.58, SD = 13), and included 12 physicians, 9 psychologists, 1 nurse, 1 biologist, and 1 researcher. Half of the CP specialists reported more than 10 years of experience in the field of CP, while the remaining 50% had between 5 and 10 years of experience. Full demographic and clinical characteristics are provided in Supporting Information (available here).

In the administration of the original NEQ to Sample A, 10 items did not reach the minimum content validity threshold. However, these items were readministered to Sample B, along with the additional items proposed by participants, to confirm the results. This initial phase led to minor revisions in the wording of two items. Specifically, in Item 9 (“I need some of my disorders to be better controlled”), the examples of symptoms in parentheses were modified: *pain* was removed, while *low mood* and *fatigue* were added. In Item 15, a reference to *social security information* was included to better capture patients’ informational needs. Additionally, several other unmet needs were identified through participants’ feedback. These were synthesized by the authors, following the procedure described in the section “Methods,” into 13 new items aimed at capturing needs relevant in the CP field but not assessed by the original NEQ. The questionnaire administered to Sample B thus consisted of 40 items.

The group of expert patients in Sample B (*n* = 14) consisted predominantly of females (93%, *N* = 13), with ages ranging from 36 to 69 years (mean age = 52.43, SD = 9.06). The majority of this group was employed (64.3%) and reported experiencing pain for more than 10 years (85.2%). The group of CP specialists in Sample B (*n* = 11) was gender‐balanced (45.5% female, 54.5% male), with ages ranging from 31 to 68 years (mean = 50.64, SD = 13.17), and consisted of 6 physicians, 4 psychologists, and 1 biologist. Regarding experience in CP care, 8 CP specialists in Sample B reported more than 10 years of experience, while 6 had between 5 and 10 years. Full demographic and clinical characteristics are provided in Supporting Information (available here).

In the administration of Sample B, the same 10 items that did not reach the minimum content validity in the first administration showed again indices of comprehensibility (I‐CI), content validity (I‐CVI), or both below the expected cutoff of 0.78 (Table 1). Thus, these items were considered inadequate and excluded. All 13 newly developed items reached the threshold for both comprehensibility and content validity and were therefore included in the preliminary version of the NEQ‐CP to be further validated. The resulting preliminary version of the NEQ‐CP consisted of 30 items.

**TABLE 1:** Comprehensibility and relevance (content validity) for the items of the *Need Evaluation Questionnaire–Chronic Pain* (NEQ‐CP).

Item	Sample A (*N* = 59)		Sample B (*N* = 25)	
	I‐CI	I‐CVI	I‐CI	I‐CVI
1. I need to have more information about my diagnosis	0.91	0.90	0.84	0.92
2. I need to have more information about my future conditions	0.85	0.88	0.92	0.96
3. I need to have more information about the diagnostic tests they are doing on me	0.91	0.90	0.84	0.84
4. I need to have more explanations about the treatments	0.96	0.93	0.92	0.92
5. I need to be more involved in the therapeutic choices	0.89	0.85	0.96	0.80
6. I need the doctors and nurses to give me more understandable information	0.90	0.89	0.96	0.92
**7. I need the doctors to be more honest with me**	**0.76**	**0.68**	0.88	**0.68**
8. I need to have more dialogue with the doctors	0.92	0.91	0.92	0.92
9. I need some of my disorders (tiredness, low mood, nausea, insomnia, etc.) to be better controlled	0.95	0.89	0.96	0.92
**10. I need more help with eating, getting dressed, and going to the bathroom**	0.87	**0.67**	0.92	**0.72**
**11. I need more respect for my privacy**	**0.69**	**0.61**	0.80	**0.56**
**12. I need more respect from the nursing staff**	0.79	**0.64**	0.84	**0.64**
**13. I need to be more reassured by the doctors**	0.86	**0.74**	0.84	**0.75**
**14. I need the services offered by the hospital (bathrooms, meals, cleaning) to be better**	0.87	**0.69**	0.96	**0.72**
15. I need to have more economic, social security, and insurance information related to my illness	0.85	0.85	0.80	0.80
16. I need financial help	0.81	0.78	0.84	0.84
17. I need to talk to a psychologist	0.92	0.89	0.88	0.96
**18. I need to talk to a spiritual assistant**	0.80	**0.52**	0.92	**0.64**
19. I need to talk to people who have had the same experience as me	0.93	0.88	0.92	0.72
20. I need to be more reassured by my family	0.86	0.77	0.92	0.80
21. I need to feel more useful in the family	0.82	0.78	0.88	0.88
22. I need to feel less abandoned to myself	0.81	0.88	0.88	0.80
**23. I need to be less pitied by others**	0.79	**0.68**	0.84	**0.68**
**24. I need help in transferring from home to the hospital**	0.86	**0.75**	0.96	0.84
25. I need there to be more dialogue between the hospital doctors and my “family doctor”	0.94	0.92	0.96	0.92
**26. I need help in dealing with problems in the sexual sphere**	0.85	**0.71**	0.96	**0.76**
27. I need more help in maintaining, as much as possible, my normal daily activities	0.89	0.91	0.92	0.92
28. I need the health professionals who follow me (pain therapy specialist, family doctor, physiotherapists, psychologists, etc.) to talk to each other more	—	—	0.92	0.96
29. I need health professionals who are more trained in pain therapy	—	—	0.92	0.92
30. I need to be followed by a single pain therapist, for greater continuity in the care relationship	—	—	0.84	0.80
31. I need my pain condition to allow me to book visits and tests with shorter waiting times	—	—	0.88	0.88
32. I need pain‐induced disability to be more recognized in the world of work	—	—	0.96	0.96
33. I need to receive exemptions for tests and medical treatments for my pain condition	—	—	0.92	0.92
34. I need pain therapy centers closer to where I live	—	—	0.92	0.88
35. I need to have more information about pain therapy centers and specialists who can help me with chronic pain	—	—	0.96	0.96
36. I need more scientific research on chronic pain to be carried out	—	—	0.88	0.80
37. I need home care			0.92	0.80
38. I need to feel more understood by others (family, coworkers, health professionals)	—	—	0.92	0.80
39. I need training sessions on chronic pain	—	—	0.84	0.80
40. I need to have a telephone contact for the pain therapy group that follows me for greater continuity in the care relationship	—	—	0.90	0.85

*Note:* Item indices < 0.78 required further analysis of the item. Scale indices 0.80–0.89 were considered good and > 0.90 as excellent [30]. Excluded items are in bold.

Abbreviations: I*-*CI, item comprehensibility indices; I‐CVI, item content validity indices.

### 3.2. Validation of the NEQ‐CP

After a preliminary analysis of 521 questionnaires received, 18 cases were found with more than 10% of missing responses in the questionnaire under development and thus eliminated from the dataset (valid response rate of 96.5%). The final number of participants was 503. Detailed sociodemographic and clinical information is given in Table 2.

**TABLE 2:** Sociodemographic and clinical descriptives of the sample (*n* = 503).

	*N*	%
Gender		
Male	33	6.6
Female	470	93.4
Age		
18–30	124	24.7
31–40	116	23.1
41–50	108	21.5
51–60	93	18.5
> 60	61	12.2
Housing situation		
Living alone	72	14.3
Living with parents	122	24.3
Living with current family	264	52.5
Living with other nonfamily people	45	8.9
Diagnosis		
Joint pain	32	6.9
Migraine/headache	18	3.9
Fibromyalgia	157	33.8
Vulvodynia	61	13.1
Back pain	17	3.7
Endometriosis	30	6.5
Neuropathic pain	39	8.4
Other	34	7.3
Comorbidity	76	16.4
Pain duration (years) (from CPG)		
Less than 1	21	4.2
From 1 to 3	103	20.5
From 4 to 5	62	12.3
From 6 to 10	110	21.9
More than 10	207	41.2
Treatment modality (from CP‐QUEST)		
Continuous therapy	342	68.1
Cyclical therapy	66	13.1
On‐demand therapy	69	13.7
No therapy	25	5.0
Educational level		
Secondary school	72	14.3
High school	240	47.8
University	142	28.3
Postgraduate	48	9.6
Occupation		
Worker	274	54.5
Unemployed	79	15.7
Housekeeper	43	8.5
Student	55	10.9
Retired	52	10.3
Place of residence		
Northern Italy	249	49.7
Central Italy	151	30.1
Southern Italy	101	20.2
Pain intensity (from CP‐QUEST)		
Very mild	1	0.2
Mild	13	2.6
Moderate	158	31.5
Severe	233	46.5
Very severe	96	19.2
Perceived treatment effectiveness (from CP‐QUEST)		
Effective treatment	29	5.8
Partially effective treatment	339	67.4
No effectiveness	115	22.8

#### 3.2.1. Descriptive Item Analysis

Percentages of affirmative answers ranged from 16% to 99% (Table 3). Three items had a percentage of affirmative answers ≥ 95%, indicating that they were not informative (all the respondents chose the yes option). Conversely, we observed only 16% of affirmative answers for Item 27. This means that the need described (“I need home care”) is perceived to a very low extent. Thus, we decided to exclude these four items to proceed with the following analyses (Table 3).

**TABLE 3:** Percentage of affirmative answers, factor loadings, chi‐square fit statistic, item discrimination (*a*), and location (*b*) estimates of items of the *Needs Evaluation Questionnaire for Chronic Pain* (NEQ‐CP).

Item	Yes (%)	*λ*	*χ* ^2^	d*f*	*p*	*a*	*B*
**1. I need to have more information about my diagnosis**	72	0.60	13.77	13	0.39	1.73	−0.80
**2. I need to have more information about my future conditions**	91	0.66	14.22	15	0.51	1.84	−1.81
**3. I need to have more information about the diagnostic tests they are doing on me**	57	0.68	9.19	9	0.42	2.57	−0.23
**4. I need to have more explanations about the treatments**	72	0.71	17.37	10	0.07	2.98	−0.67
**5. I need to be more involved in the therapeutic choices**	69	0.77	7.66	8	0.47	3.74	−0.56
**6. I need the doctors and nurses to give me more understandable information**	66	0.68	15.57	11	0.16	2.38	−0.52
**7. I need to have more dialogue with the doctors**	81	0.76	12.55	13	0.48	2.23	−1.09
**8. I need some of my disorders (tiredness, nausea, insomnia, etc.) to be better controlled**	93	0.74	8.68	13	0.80	1.82	−2.10
**9. I need to have more economic, social security, and insurance information related to my illness**	90	0.68	4.28	14	0.99	1.56	−1.94
10. I need financial help	72	0.48	Excluded (*a* < 0.65)				
**11. I need to talk to a psychologist**	75	0.47	20.06	16	0.22	0.66	−1.82
**12. I need to talk to people who have had the same experience as me**	85	0.47	20.77	16	0.19	0.91	−2.15
13. I need to be more reassured by my family	63	0.49	Excluded (LD > 10)				
14. I need to feel more useful in the family	64	0.41	Excluded (*a* < 0.65)				
**15. I need to feel less abandoned to myself**	79	0.61	9.94	15	0.82	1.07	−1.52
16. I need there to be more dialogue between the hospital doctors and my “family doctor”	75	0.66	Excluded (LD > 10)				
**17. I need more help in maintaining, as much as possible, my normal daily activities**	77	0.59	12.78	15	0.62	0.89	−1.54
**18. I need the health professionals who follow me (pain therapy specialist, family doctor, physiotherapists, psychologists, etc.) to talk to each other more**	85	0.71	9.91	15	0.83	1.52	−1.57
**19. I need health professionals who are more trained in pain therapy**	87	0.74	13.99	14	0.45	1.86	−1.53
**20. I need to be followed by a single pain therapist, for greater continuity in the care relationship**	73	0.51	12.31	15	0.66	0.97	−1.22
21. I need my pain condition to allow me to book visits and tests with shorter waiting times	95		Excluded (% ≥ 95)				
22. Need pain‐induced disability to be more recognized in the world of work	96		Excluded (% ≥ 95)				
**23. I need to receive exemptions for tests and medical treatments for my pain condition**	94	0.63	11.55	14	0.64	1.35	−2.54
24. I need pain therapy centers closer to where I live	72	0.52	Excluded (LD > 10)				
**25. I need to have more information about pain therapy centers and specialists who can help me with chronic pain**	86	0.66	13.39	14	0.48	1.51	−1.60
26. I need more scientific research on chronic pain to be carried out	99		Excluded (% ≥ 95)				
27. I need home care	16		Excluded (low %)				
**28. I need to feel more understood by others (family, coworkers, health professionals)**	86	0.61	25.71	16	0.06	1.01	−2.08
**29. I need training sessions on chronic pain**	73	0.57	12.69	15	0.62	1.16	−1.08
**30. I need to have a telephone contact for the pain therapy group that follows me for greater continuity in the care relationship**	66	0.58	8.99	14	0.83	1.14	−0.71

*Note:* Parameters were computed under the 2PL model (*a* = discrimination, *b* = location). The items of the NEQ‐CP in its final version are in bold.

Abbreviations: 2PL, two‐parameter model; d*f*, degrees of freedom.

#### 3.2.2. IRT Analysis

As prerequisites, dimensionality and local dependence were tested. Prior to conducting the EFA, Bartlett’s test of sphericity was performed. The null hypothesis that the population tetrachoric correlation matrix is an identity matrix was rejected (*χ* ^2^ = 22527.034, d*f* = 325, *p* < 0.001), supporting the factorability of the data. An exploratory factor analysis was conducted on the tetrachoric correlation matrix using the principal axis factoring (PAF) extraction method. Results indicated that a single factor explained 39% of the variance, supporting a unidimensional structure. Factor loadings ranged from 0.41 to 0.77 (Table 3). The full tetrachoric correlation matrix of the final 21 items is reported in Supporting Information (available here). LD statistics were > 10 for three couples of items (13 and 28, 16 and 18, 24 and 25). Looking at the content of the item, we found that it was very similar. To avoid redundancy, Item 13, Item 16, and Item 24 were removed. Then, the prerequisites for IRT analysis were met.

**FIGURE 1:**
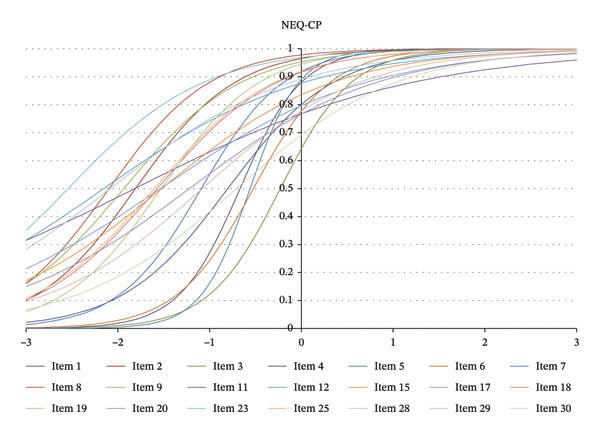
Item characteristic curves (ICCs) of each item of the *Needs Evaluation Questionnaire for Chronic Pain* (NEQ‐CP). Latent trait (Theta) is shown on the horizontal axis. The probability of item endorsement is shown on the vertical axis.

The 2PL model was tested. At a first step, all the items have an adequate fit (*p* < 0.02, *α* adjusted for multiple comparisons: 0.05/23 = 0.002). Item 14 (“I need to feel more useful in the family”) showed low discrimination (0.64). Thus, it was excluded. The following analysis revealed that all the items show an adequate fit (*p* < 0.05), but Item 10 (“I need financial help”) had poor discrimination ability (0.64). Thus, this item was also excluded. IRT parameters of the final NEQ‐CP are reported in Table 2. All the items have an adequate fit (*p* < 0.05). The *b* parameters are spaced along the negative values of the trait (range from −2.54 to −0.23). Lower values identify needs perceived to a higher extent (e.g., Item 23: “I need to receive exemptions for tests and medical treatments for my pain condition” and Item 12: “I need to talk to people who have had the same experience as me”), while lower values represent less compelling needs (e.g., Item 3: “I need to have more information about the diagnostic tests they are doing on me” and Item 6: “I need the doctors and nurses to give me more understandable information”). The *a* parameter represents the discrimination ability of the item. The higher the *a*, the better the item’s ability to differentiate between people with different levels of unmet needs. The discrimination values (*a*) ranged from 0.66 to 3.74. According to Baker and Kim [51], eight items showed moderate, four high, and the remaining nine items very high discriminative power (Table 2). Figure 1 graphically represents the NEQ‐CP item location along the trait continuum and discrimination ability, represented by the steepness of the curves.

#### 3.2.3. Reliability

**FIGURE 2:**
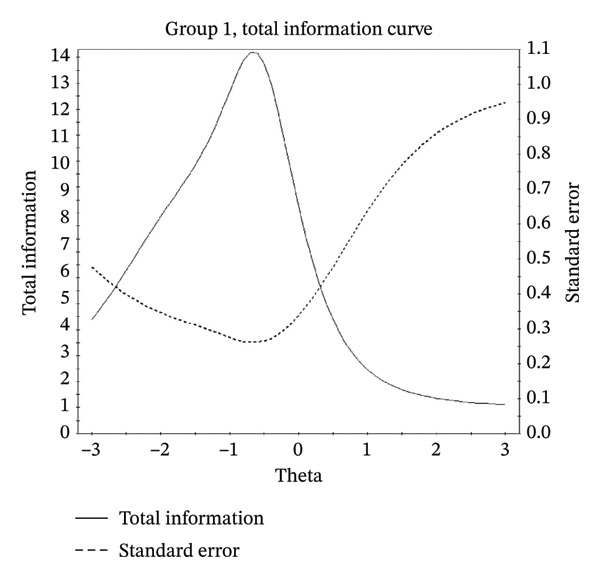
Test information function of the *Needs Evaluation Questionnaire for Chronic Pain* (NEQ‐CP). Latent trait (Theta) is shown on the horizontal axis. The amount of information (solid line) and the standard error (dotted line) yielded by the test at any trait level are shown on the vertical axis.

The NEQ‐CP showed good reliability (KR‐20 = 0.856 [CI 90%: 0.837–0.874]). The test information curve shows the local reliability (Figure 2). Within a large range of traits (from −3.00 to 0.50), the amount of test information was greater than 4, indicating that the NEQ‐CP was highly informative. Indeed, if we convert the information in the associated reliability (*r* = 1‐1/Information), the reliability was equal to or greater than 0.75 within this range. Since summed scores of the questionnaire (i.e., the number of unmet needs reported by the patient) can be translated into IRT trait scores, we can observe that the NEQ‐CP is very reliable for a wide score range (precisely, from 0 to 12 unmet needs).

#### 3.2.4. Validity

As expected, the NEQ‐CP scores were positively correlated with pain intensity (*r* = 0.23, *p* < 0.01) and pain interference (*r* = 0.42, *p* = 0.50). No correlation emerged between the NEQ‐CP score and the duration of pain (*r* = 0.04, *p* < 0.01). Positive correlations were found with anxiety, depression, and stress. Additionally, the NEQ‐CP score was negatively related to subjective perception of mental and physical health, and to all the well‐being dimensions (Table 4).

**TABLE 4:** Bivariate correlates between the *Needs Evaluation Questionnaire for Chronic Pain* (NEQ‐CP), subjective perception of mental and physical health, depression, stress, anxiety, and well‐being.

	Needs evaluation questionnaire for chronic pain (NEQ‐CP)
1. Mental health score (MCS‐12)	−0.47^∗∗^
2. Physical health score (PCS‐12)	−0.35^∗∗^
3. Depression anxiety stress scale–depression (DASS‐D)	0.43^∗∗^
4. Depression anxiety stress scale–stress (DASS‐S)	0.38^∗∗^
5. Depression anxiety stress scale–anxiety (DASS‐A)	0.39^∗∗^
6. Numerical rating scale–physical (WB‐NRS1)	−0.31^∗∗^
7. Numerical rating scale–psychological (WB‐NRS2)	−0.40^∗∗^
8. Numerical rating scale–relational (WB‐NRS3)	−0.35^∗∗^
9. Numerical rating scale–spiritual (WB‐NRS4)	−0.28^∗∗^
10. Numerical rating scale–general (WB‐NRS5)	−0.37^∗∗^

*Note:* Values represent Pearson’s correlation coefficients (*r*).

^∗∗^Correlation is significant at *p* < 0.01 (2‐tailed).

**FIGURE 3:**
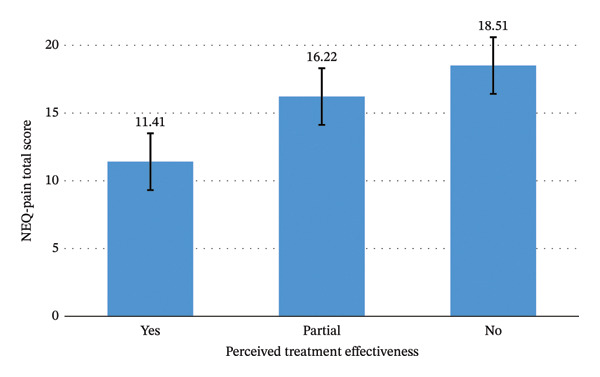
Differences in the *Needs Evaluation Questionnaire for Chronic Pain* (NEQ‐CP) score among patients with different levels of therapy efficacy (yes, partial, no).

Patients not receiving therapy reported higher unmet needs compared to those receiving therapy (*p* < 0.01, *d* = 0.54). In addition, unmet needs differed according to perceived treatment effectiveness (*p* < 0.001, ηp^2^ = 0.14), with lower scores among patients reporting effective treatments and higher levels among those reporting partial or no effectiveness (see Figure 3). No significant differences in NEQ‐CP scores were observed across sociodemographic and clinical variables, including age, educational level, occupation, housing situation, and diagnosis (all *p* > 0.05), with small or negligible effect sizes. This suggests that unmet needs are broadly distributed across different subgroups of patients with CP and are not strongly influenced by these characteristics.

## 4. Discussion

The high prevalence, diagnostic–therapeutic challenges, and severe consequences have led various stakeholders to declare CP as a public health priority worldwide [3, 54, 55]. Hence, to meet the need of improving services and support, the current study aimed to develop a scale to evaluate the unmet needs of CP patients. Starting from modifying the NEQ [24, 27, 56], WE OBTAINED THE NEQ‐CP, a disease‐specific instrument that includes general items along with items tailored to CP.

The current questionnaire was developed by employing IRT. One advantage of IRT is that it provides unbiased estimates of item parameters even when a sample is unrepresentative [52]. As such, whereas the current sample was unbalanced by gender and diagnosis, using IRT, we were able to provide evidence of the questionnaire’s suitability. The spread of location parameters indicated that most needs were perceived as compelling by CP patients. Additionally, the large discriminative power observed for several items supported the reliability of the scale.

Validity measures supported the expected negative relationships between well‐being and the individual’s subjective perception of physical and mental health, while anxiety and depression were positively correlated with the number of unmet needs. These correlations can be interpreted as indices of the discriminant construct validity of the questionnaire because they are significant but of medium magnitude. That is, these constructs are theoretically related to but different from the needs measured by the NEQ‐CP. These results strongly support the importance of assessing the unmet needs of CP patients, as it helps to pay more attention to specific requests and provide solutions when possible. Examining unmet needs may indicate the necessity of starting intervention programs to ensure they are sufficiently addressed. Furthermore, the relationships with well‐being, depression, and anxiety can also be taken as evidence of criterion validity by considering the NEQ‐CP as a predictor. Therefore, to prevent or reduce depression and anxiety and promote well‐being, it is important to be aware of unmet needs and find appropriate clinical practice standards to support CP patients. Finally, evidence of validity was provided observing that higher pain intensity and pain interference were associated with a larger number of needs. Consistently, a significant difference in unmet needs was observed depending on receiving pain treatments and the perceived outcomes of the therapy (i.e., successful, partially beneficial, and no efficacy).

Broadly speaking, empirical evidence that the NEQ‐CP is psychometrically sound supports both its effectiveness and use in detecting patients who express a significant number of unmet needs and, therefore, may receive targeted answers (e.g., information on diagnosis, therapy, and symptom control). According to relevant COSMIN recommendations for patient‐reported outcome measures, the present study provides evidence for content validity, structural validity, internal consistency, and construct validity of the NEQ‐CP. However, given the cross‐sectional design, additional measurement properties such as test–retest reliability, measurement error, and responsiveness remain to be established in future research. Collecting information at the individual level makes it possible to give effective answers in solving specific problems and to identify individuals who need prompt support. At the same time, NEQ‐CP can be used in research surveys to collect information that allows health policymakers to allocate available resources where the needs are greater and more urgent, as well as to assess the effectiveness of targeted interventions after their implementation.

Referring to the use of the NEQ‐CP as a survey instrument, the current study highlights high percentages of unmet needs among CP individuals, often exceeding those reported in other chronic conditions. For example, compared to data reported for patients with cancer, head and neck cancer, and chronic liver disease [24, 57, 58] (Bonacchi et al., 2022), our CP participants showed a greater need for effective symptom management. Even more concerning was the gap in the need for psychological support. Three‐quarters of the sample expressed the need to consult with a psychologist, while this need was expressed by less than a fifth of cancer and liver disease patients [24, 57, 59]. Overall, the current data collection suggests that an overwhelming majority of CP participants called for recognition of CP‐related disability, echoing concerns raised by advocacy groups and national reports [60, 61].

This research has some limitations to consider. A key limitation lies in the inherent complexity and evolving nature of unmet needs in CP patients, which may not be fully captured by a structured, fixed‐response questionnaire. While qualitative methods such as interviews offer in‐depth insights, the use of a psychometrically validated scale allows for systematic assessment across larger samples and settings, supporting comparisons and informing service planning. Importantly, the development of the NEQ‐CP was grounded in qualitative input from both patients and CP specialists, and additional items were derived from their open‐ended responses. Future research should combine quantitative and qualitative approaches to better reflect the full complexity of unmet needs.

Another limitation concerns the representativeness of the sample. The use of an online recruitment strategy may have introduced selection bias, as individuals who are younger, more educated, and with greater access to digital resources are more likely to participate. This is reflected in our sample, which appears younger, more highly educated, and markedly unbalanced in terms of gender, with a higher proportion of female participants compared to epidemiological data on CP in both European [62] and Italian populations [4]. Moreover, the distribution of clinical and sociodemographic characteristics differs from population‐based studies. Compared to Italian representative data [4], our sample shows a lower prevalence of mild or very mild pain (2.8% vs. 18.2%), a higher prevalence of continuous CP treatment (68.1% vs. 32.3%), a greater proportion of younger individuals (24.7% vs. 7%), a markedly higher proportion of female participants (93.4% vs. 60.7%), and a higher level of education (37.9% vs. 9.4%). Additionally, specific conditions such as fibromyalgia appear to be overrepresented. These differences likely reflect the recruitment method and should be considered when interpreting the generalizability of the findings. As mentioned, the current questionnaire was developed by employing IRT. An advantage of the IRT approach is that it can provide unbiased estimates of item parameters even when a sample is unrepresentative [52]. This invariance property of IRT states that item parameters are independent of the sample and, as a consequence, they are invariant with respect to the sample characteristics from which they are generated. As such, whereas the current sample was unbalanced by gender, age, pain levels, and diagnosis, using IRT we were able to provide evidence of the questionnaire’s suitability. Nonetheless, the findings obtained should be generalized with caution, and future studies should confirm and extend the current results, addressing such limitations. Particularly, collecting data on more distributed samples across genders, age, and diagnosis will allow for performing differential item functioning (DIF) analyses to test the measurement equivalence of the NEQ‐CP across male and female, younger, and older patients, and different CP diseases. Moreover, to extend the use of the current scale, it would be relevant to test the psychometric properties of different language versions of the scale and to use DIF analysis across languages to provide evidence of the invariance property of the scale.

Furthermore, future longitudinal studies should evaluate the test–retest reliability of the NEQ‐CP to establish the temporal stability of the instrument, as well as its responsiveness to change (e.g., in assessing the efficacy of intervention programs targeting patients’ unmet needs).

Finally, future studies should incorporate additional psychological measures commonly used in CP research, such as the Pain Catastrophizing Scale, to further elucidate the relationship between cognitive–emotional processes and perceived unmet needs. Moreover, future surveys employing the NEQ‐CP could benefit from including more detailed socioeconomic indicators such as annual income, social deprivation indices, and access to healthcare services. Collecting these variables will be essential for identifying the structural and social factors that contribute to variations in patients’ perceived unmet needs.

In conclusion, the NEQ‐CP has the potential to be an effective and easy‐to‐use tool in the assessment of the unmet needs of patients with CP, particularly in survey‐based research and routine clinical practice, where it may support both large‐scale evaluations and the optimization of patient‐centered care at individual and service levels. From an administration perspective, the NEQ‐CP is designed to use a dichotomous (yes/no) response format, allowing rapid identification of unmet needs. Although some items may be conceptually related to common thematic areas, the scale was developed using an IRT approach and is not structured into formally validated subscales. Therefore, presenting items in a mixed order may be preferable to reduce potential response biases, such as halo effects, and to promote independent evaluation of each need.

## Author Contributions

Francesca Chiesi, Michael Tenti, and Andrea Bonacchi conceived the study and contributed to its design. Material preparation and data collection were performed by Michael Tenti. Data analysis was performed by Francesca Chiesi, Costanza Gori, Caterina Primi, and Carlotta Tagliaferro. Francesca Chiesi, Costanza Gori, Caterina Primi, and Michael Tenti wrote and edited the paper. All the authors discussed the results and commented on the manuscript.

## Funding

No funding was received for this manuscript.

Open access publishing was facilitated by Universita degli Studi di Firenze, as part of the Wiley–CRUI‐CARE agreement.

## Conflicts of Interest

The authors declare no conflicts of interest.

## Supporting Information

Additional supporting information can be found online in the Supporting Information section.

## Supporting information


**Supporting Information 1** Supporting file 1: Full demographic and clinical characteristics of the two groups involved in the development of the preliminary version of the Needs Evaluation Questionnaire–Chronic Pain (NEQ‐CP): Group A (“group of CP specialists”) and Group B (“group of expert patients”).

**Supporting Information 2** Supporting file 2: Full tetrachoric correlation matrix of the final 21 items of the Needs Evaluation Questionnaire–Chronic Pain (NEQ‐CP).

## Data Availability

The data that support the findings of this study are available upon request from the corresponding author. The data are not publicly available due to privacy or ethical restrictions.

## References

[1] Treede R. D. , Rief W. , Barke A. et al., A Classification of Chronic Pain for ICD-11, Pain. (2015) 156, no. 6, 1003–1007, 10.1097/j.pain.0000000000000160. 25844555 PMC4450869

[2] Rafaeli W. , Tenti M. , Corraro A. et al., Chronic Pain: What Does It Mean? A Review on the Use of the Term Chronic Pain in Clinical Practice, Journal of Pain Research. (2021) 14, 827–835. 33833560 10.2147/JPR.S303186 PMC8019660

[3] Goldberg D. S. and McGee S. J. , Pain as a Global Public Health Priority, BMC Public Health. (2011) 11, 1–5, 10.1186/1471-2458-11-770. 21978149 PMC3201926

[4] Maraschini A. , Tenti M. , Raffaeli W. et al., Chronic Pain Prevalence and Psychosocial Burden in the Italian Population from the 2019 European Health Interview Survey, International Journal of Environmental Research and Public Health. (2025) 22, no. 9, 10.3390/ijerph22091395. PMC12469650 41007538

[5] Dueñas M. , Ojeda B. , Salazar A. , Mico J. A. , and Failde I. , A Review of Chronic Pain Impact on Patients, Their Social Environment and the Health Care System, Journal of Pain Research. (2016) 9, 457–467, 10.2147/JPR.S105892. 27418853 PMC4935027

[6] Breivik H. , Collett B. , Ventafridda V. , Cohen R. , and Gallacher D. , Survey of Chronic Pain in Europe: Prevalence, Impact on Daily Life, and Treatment, European Journal of Pain. (2006) 10, no. 4, 287–333, 10.1016/j.ejpain.2005.06.009. 16095934

[7] Carnago L. , O′Regan A. , and Hughes J. M. , Diagnosing and Treating Chronic Pain: Are We Doing This Right?, Journal of Primary Care & Community Health. (2021) 12, 10.1177/21501327211008055. PMC8072853 33882736

[8] Newton B. J. , Southall J. L. , Raphael J. H. , Ashford R. L. , and LeMarchand K. , A Narrative Review of the Impact of Disbelief in Chronic Pain, Pain Management Nursing. (2013) 14, no. 3, 161–171, 10.1016/j.pmn.2010.09.001. 23972867

[9] Tenti M. , Raffaeli W. , Paroli M. et al., An Italian Survey and Focus Groups on Fibromyalgia Impairment: Impact on Work and Possible Reasonable Accommodations, Healthcare. (2024) 12, no. 2, 10.3390/healthcare12020216. PMC10815387 38255103

[10] Carey M. , Lambert S. , Smits R. , Paul C. , Sanson-Fisher R. , and Clinton-McHarg T. , The Unfulfilled Promise: A Systematic Review of Interventions to Reduce the Unmet Supportive Care Needs of Cancer Patients, Supportive Care in Cancer. (2012) 20, no. 2, 207–219, 10.1007/s00520-011-1327-1. 22089430 PMC3244607

[11] Osse B. H. , Vernooij-Dassen M. J. , de Vree B. P. , Schadé E. , and Grol R. P. , Assessment of the Need for Palliative Care as Perceived by Individual Cancer Patients and Their Families: A Review of Instruments for Improving Patient Participation in Palliative Care, Cancer. (2000) 88, no. 4, 900–911, 10.1002/(sici)1097-0142(20000215)88:4<900::aid-cncr22>3.0.co;2-2. 10679661

[12] Willems R. A. , Bolman C. A. , Mesters I. , Kanera I. M. , Beaulen A. A. , and Lechner L. , Cancer Survivors in the First Year After Treatment: The Prevalence and Correlates of Unmet Needs in Different Domains, Psycho-Oncology. (2016) 25, no. 1, 51–57, 10.1002/pon.3870. 26110652

[13] Davis C. , Williams P. , Redman S. , White K. , and King E. , Assessing the Practical and Psychosocial Needs of Rural Women With Early Breast Cancer in Australia, Social Work in Health Care. (2002) 36, no. 3, 25–36, 10.1300/j010v36n03_02. 12564650

[14] Karra R. , Holten-Rossing S. , Mohammed D. , Parmeggiani L. , Heine M. , and Namnún O. C. , Unmet Needs in the Management of Functional Impairment in Patients With Chronic Pain: A Multinational Survey, Pain Management. (2021) 11, no. 3, 303–314, 10.2217/pmt-2020-0098. 33353407

[15] Sanders C. , Donovan J. L. , and Dieppe P. A. , Unmet Need for Joint Replacement: A Qualitative Investigation of Barriers to Treatment Among Individuals With Severe Pain and Disability of the Hip and Knee, Rheumatology. (2004) 43, no. 3, 353–357. 14623947 10.1093/rheumatology/keh044

[16] McCarberg B. H. , Nicholson B. D. , Todd K. H. , Palmer T. , and Penles L. , The Impact of Pain on Quality of Life and the Unmet Needs of Pain Management: Results From Pain Sufferers and Physicians Participating in an Internet Survey, American Journal of Therapeutics. (2008) 15, no. 4, 312–320, 10.1097/mjt.0b013e31818164f2. 18645331

[17] Boulton T. , Nothing and Everything: Fibromyalgia as a Diagnosis of Exclusion and Inclusion, Qualitative Health Research. (2019) 29, no. 6, 809–819, 10.1177/1049732318804509. 30296924

[18] Jeppesen M. M. , Mogensen O. , Dehn P. , and Jensen P. T. , Needs and Priorities of Women With Endometrial and Cervical Cancer, Journal of Psychosomatic Obstetrics and Gynecology. (2015) 36, no. 3, 122–132, 10.3109/0167482x.2015.1059417. 26123123

[19] Phillips R. , Pell B. , Grant A. et al., Identifying the Unmet Information and Support Needs of Women With Autoimmune Rheumatic Diseases During Pregnancy Planning, Pregnancy and Early Parenting: Mixed-Methods Study, BMC Rheumatology. (2018) 2, no. 1, 1–18, 10.1186/s41927-018-0029-4. 30886972 PMC6390539

[20] Stobbe J. , Wierdsma A. I. , Kok R. M. , Kroon H. , Depla M. , and Mulder C. L. , Decrease in Unmet Needs Contributes to Improved Motivation for Treatment in Elderly Patients With Severe Mental Illness, Social Psychiatry and Psychiatric Epidemiology. (2015) 50, no. 1, 125–132, 10.1007/s00127-014-0918-9. 24985314

[21] Moreno P. I. , Ramirez A. G. , San Miguel-Majors S. L. et al., Unmet Supportive Care Needs in Hispanic/Latino Cancer Survivors: Prevalence and Associations With Patient-Provider Communication, Satisfaction With Cancer Care, and Symptom Burden, Supportive Care in Cancer. (2019) 27, no. 4, 1383–1394, 10.1007/s00520-018-4426-4. 30136022 PMC6386634

[22] Stomski N. J. , Mackintosh S. , and Stanley M. , Patient Self-Report Measures of Chronic Pain Consultation Measures: A Systematic Review, The Clinical Journal of Pain. (2010) 26, no. 3, 235–243, 10.1097/ajp.0b013e3181c84e76. 20173438

[23] Letzen J. E. , Hunt C. A. , Webb C. et al., Preliminary Validation of the Pain Relief Motivation Scales, The Clinical Journal of Pain. (2024) 40, no. 1, 46–56, 10.1097/ajp.0000000000001170. 37921577 PMC10841969

[24] Tamburini M. , Gangeri L. , Brunelli C. et al., Assessment of Hospitalised Cancer Patients’ Needs by the Needs Evaluation Questionnaire, Annals of Oncology. (2000) 11, no. 1, 31–38, 10.1023/a:1008396930832. 10690384

[25] Tamburini M. , Gangeri L. , Brunelli C. et al., Cancer Patients’ Needs During Hospitalisation: A Quantitative and Qualitative Study, BMC Cancer. (2003) 3, no. 1, 1–11, 10.1186/1471-2407-3-12. 12710890 PMC155542

[26] Annunziata M. A. , Muzzatti B. , and Altoè G. , A Contribution to the Validation of the Needs Evaluation Questionnaire (NEQ): A Study in the Italian Context, Psycho‐Oncology: Journal of the Psychological, Social and Behavioral Dimensions of Cancer. (2009) 18, no. 5, 549–553, 10.1002/pon.1445. 19021128

[27] Bonacchi A. , Miccinesi G. , Galli S. et al., Use of the Needs Evaluation Questionnaire With Cancer Outpatients, Supportive Care in Cancer. (2016) 24, no. 8, 3507–3515, 10.1007/s00520-016-3176-4. 27005464

[28] Tsang S. , Royse C. F. , and Terkawi A. S. , Guidelines for Developing, Translating, and Validating a Questionnaire in Perioperative and Pain Medicine, Saudi Journal of Anaesthesia. (2017) 11, no. Suppl 1, S80–S89, 10.4103/sja.sja_203_17. 28616007 PMC5463570

[29] Streiner D. L. and Kottner J. , Recommendations for Reporting the Results of Studies of Instrument and Scale Development and Testing, Journal of Advanced Nursing. (2014) 70, no. 9, 1970–1979, 10.1111/jan.12402. 24684713

[30] Polit D. F. , Beck C. T. , and Owen S. V. , Is the CVI an Acceptable Indicator of Content Validity? Appraisal and Recommendations, Research in Nursing & Health. (2007) 30, no. 4, 459–467, 10.1002/nur.20199. 17654487

[31] DeVellis R. F. , Scale Development: Theory and Applications, 2017, 4th edition, Sage.

[32] Kyriazos T. A. , Applied Psychometrics: Sample Size and Sample Power Considerations in Factor Analysis (EFA, CFA) and SEM in General, Psychology. (2018) 9, no. 8, 2207–2230, 10.4236/psych.2018.98126.

[33] Toccaceli V. , Tenti M. , Stazi M. A. et al., Development and Validation of the Italian “Brief Five-Item Chronic Pain Questionnaire” for Epidemiological Studies, Journal of Pain Research. (2022) 15, 1897–1913, 10.2147/jpr.s362510. 35837542 PMC9275508

[34] Salafi F. , Stancati A. , and Grassi W. , Reliability and Validity of the Italian Version of the Chronic Pain Grade Questionnaire in Patients With Musculoskeletal Disorders, Clinical Rheumatology. (2006) 25, no. 5, 619–631. 16421646 10.1007/s10067-005-0140-y

[35] Ware Jr J. E. , Kosinski M. , and Keller S. D. , A 12-Item Short-Form Health Survey: Construction of Scales and Preliminary Tests of Reliability and Validity, Medical Care. (1996) 34, no. 3, 220–233, 10.1097/00005650-199603000-00003. 8628042

[36] Ware J. E. , Snow K. K. , Kosinski M. , and Gandek B. , SF-36 Health Survey, Manual and Interpretation Guide. (1993) The Health Institute, New England Medical Center, Boston, MA.

[37] Bonacchi A. , Chiesi F. , Lau C. et al., Rapid and Sound Assessment of Well-Being Within a Multi-Dimensional Approach: The Well-Being Numerical Rating Scales (WB-NRSs), PLoS One. (2021) 16, no. 6, 10.1371/journal.pone.0252709. PMC8202918 34125831

[38] Chiesi F. , Tagliaferro C. , Marunic G. , and Bonacchi A. , Measuring Spiritual Well-Being Using a Numerical Rating Scale: Additional Evidence of the Validity of the Well-Being Numerical Rating Scales (WB-NRSs), Journal of Health Psychology. (2024) 29, no. 9, 1018–1028, 10.1177/13591053231225908. 38282375

[39] Luo Q. , Liu C. , Zhou Y. et al., Chinese Cross-Cultural Adaptation and Validation of the Well-Being Numerical Rating Scales, Frontiers in Psychiatry. (2023) 14, 10.3389/fpsyt.2023.1208001. PMC10585061 37867763

[40] Chiesi F. , Share to Well: Sharing a Common Tool to Assess Well-Being at the University: Validation of Different Language Versions of the Well-Being Numerical Rating Scales (WB-NRSs), European University for Well-Being Showcase Conference: Becoming a Voice for Well-Being. (2023) University of Murcia, Murcia.

[41] Taylor R. , Lovibond P. F. , Nicholas M. K. , Cayley C. , and Wilson P. H. , The Utility of Somatic Items in the Assessment of Depression in Patients With Chronic Pain: A Comparison of the Zung Self-Rating Depression Scale and the Depression Anxiety Stress Scales in Chronic Pain and Clinical and Community Samples, The Clinical Journal of Pain. (2005) 21, no. 1, 91–100. 15599136 10.1097/00002508-200501000-00011

[42] Bottesi G. , Ghisi M. , Altoè G. , Conforti E. , Melli G. , and Sica C. , The Italian Version of the Depression Anxiety Stress Scales-21: Factor Structure and Psychometric Properties on Community and Clinical Samples, Comprehensive Psychiatry. (2015) 60, 170–181, 10.1016/j.comppsych.2015.04.005. 25933937

[43] JASP Team , JASP (Version 0.18.1), 2023, https://jasp-stats.org/.

[44] Cai L. , Du Toit S. H. C. , and Thissen D. , IRTPRO: Flexible, Multidimensional, Multiple Categorical IRT Modeling, 2011, Scientific Software International.

[45] Kline R. B. , Principles and Practice of Structural Equation Modeling, 2016, 4th edition, The Guilford Press.

[46] Reckase M. D. , Unifactor Latent Trait Models Applied to Multifactor Tests: Results and Implications, Journal of Educational Statistics. (1979) 4, no. 3, 207–230, 10.2307/1164671.

[47] Drasgow F. and Parsons C. K. , Application of Unidimensional Item Response Theory Models to Multidimensional Data, Applied Psychological Measurement. (1983) 7, no. 2, 189–199, 10.1177/014662168300700207.

[48] Jöreskog K. G. , Factor Analysis by MINRES. Technical Report, 2003, https://www.ssicentral.com/lisrel/techdocs/minres.pdf.

[49] Chen W. H. and Thissen D. , Local Dependence Indexes for Item Pairs Using Item Response Theory, Journal of Educational and Behavioral Statistics. (1997) 22, no. 3, 265–289, 10.3102/10769986022003265.

[50] Bock R. D. and Aitkin M. , Marginal Maximum Likelihood Estimation of Item Parameters: Application of an EM Algorithm, Psychometrika. (1981) 46, no. 4, 443–459, 10.1007/bf02293801.

[51] Baker F. B. and Kim S. H. , Item Response Theory: Parameter Estimation Techniques, 2004, 2nd edition, CRC Press.

[52] Embretson S. E. and Reise S. P. , Item Response Theory for Psychologists, 2000, Lawrence Erlbaum.

[53] Thiessen D. and Orlando M. , Thissen D. and Wainer H. , IRT for Items Scored in Two Categories, Test Scoring, 2001, Lawrence Earlbaum Associates, Mahwah, NJ, 73–140.

[54] Pizzo P. A. , Clark N. M. , and Carter-Pokras O. , Relieving Pain in America: A Blueprint for Transforming Prevention, Care, Education, and Research, 2011, Institute of Medicine.

[55] Hanna M. , Pain in Europe: A Public Health Priority, Journal of Pain & Palliative Care Pharmacotherapy. (2012) 26, no. 2, 182–184, 10.3109/15360288.2012.681839.

[56] Bonacchi A. , Fazzini E. , Messina S. et al., Sociodemographic, Clinical, and Psychological Characteristics Identify Groups of Italian Cancer Patients With High Rates of Unmet Needs, Tumori. (2019) 105, no. 4, 288–295, 10.1177/0300891618792458. 30185126

[57] Ferri A. , Lilloni G. , Molteni G. et al., The Psychosocial Needs of Head and Neck Cancer Patients: A Multicenter Study, European Archives of Oto-Rhino-Laryngology. (2024) 281, no. 9, 1–8, 10.1007/s00405-024-08680-3. 38704510

[58] Osowiecka K. , Szwiec M. , Dolińska A. et al., Unmet Non-Medical Needs of Cancer Patients in Poland: A Quantitative and Qualitative Study, Supportive Care in Cancer. (2024) 32, no. 3, 10.1007/s00520-024-08387-5. PMC10884169 38388767

[59] Bonacchi A. , Chiesi F. , Marunic G. et al., Needs Evaluation Questionnaire for Liver Disease: A Novel Assessment of Unmet Needs in Patients With Chronic Liver Disease, Hepatology Communications. (2023) 7, no. 2, 10.1097/hc9.0000000000000007. PMC9988321 36706170

[60] The British Pain Society , British Pain Society Press Release: Chronic Pain Costs the UK £Billions but Research Funding is Inadequate, 2018, https://www.britishpainsociety.org/mediacentre/news/british-pain-society-press-release-chronic-pain-costs-the-uk-billions-but-research-funding-is-inadequate/.

[61] Cittadinanzattiva , Presentato l’Annuale Rapporto Civico Sulla Salute: U “Fermo Immagine” Sulla Difficoltà di Accesso ai Servizi Sanitari, 2024, https://www.cittadinanzattiva.it/comunicati/16710-presentato-lannuale-rapporto-civico-sulla-salute-un-fermo-immagine-sulla-difficolta-di-accesso-ai-servizi-sanitari.html?highlight=WyJsaXN0ZSIsImRpIiwiYXR0ZXNhIl0=.

[62] Rometsch C. , Martin A. , Junne F. , and Cosci F. , Chronic Pain in European Adult Populations: A Systematic Review of Prevalence and Associated Clinical Features, Pain. (2025) 166, no. 4, 719–731, 10.1097/j.pain.0000000000003406. 40101218 PMC11921450

