# Supplementary material for: Assessing Unmet Needs of Patients With Chronic Pain: The Development and Validation of the Needs Evaluation Questionnaire for Chronic Pain
Source: Pain Res Manag. 2026 Jul 29;2026:6906748. doi: 10.1155/prm/6906748 (PMC13419345; doi:10.1155/prm/6906748)
Supplement: Supplementary file 1 — Supporting Information 1 Supporting file 1: Full demographic and clinical characteristics of the two groups involved in the development of the preliminary version of the Needs Evaluation Questionnaire–Chronic Pain (NEQ‐CP): Group A (“group of CP specialists”) and Group B (“group of expert patients”). [file PRM-2026-6906748-s002.docx]

**Supplementary materials**

**Table 1S. Socio-demographic and clinical characteristics of expert patients included in sample A (N=35).**

|  | **Mean (SD)** | **N (%)** |
| --- | --- | --- |
| **Age** | 46.86 (12.67) |  |
| **Sex**  Female  Male |  | 35 (100)  0 (0) |
| **Education**  Middle school  High school  University degree  Post-graduate education |  | 5 (14.3)  13 (37.1)  14 (40)  3 (8.6) |
| **Marital status**  Unmarried  Married  Separated/divorced |  | 11 (31.5)  20 (57.1)  4 (11.4) |
| **Occupation**  Employed  Unemployed  Housewife  Student  Retired |  | 23 (65.8)  4 (11.4)  4 (11.4)  2 (5.7)  2 (5.7) |
| **Pain duration**  From 1 to 3 years  From 4 to 10 years  From 10 to 20 years  More than 20 years |  | 2 (5.7)  10 (28.6)  11 (31.4)  12 (34.3) |

**Table 2S. Socio-demographic characteristics of CP specialists included in sample A (N=24).**

|  | **Mean (SD)** | **N (%)** |
| --- | --- | --- |
| **Age** | 49.58 (13) |  |
| **Sex**  Female  Male |  | 12 (50)  12 (50) |
| **Occupation**  Biologist  Nurse  Physician  Psychologist  Researcher |  | 1 (4.2)  1 (4.2)  12 (50)  9 (37.5)  1 (4.2) |
| **Years of experience**  5–10 years  > 10 years |  | 12 (50%)  12 (50%) |

**Table 3S. Socio-demographic and clinical characteristics of expert patients included in sample B (N=14).**

|  | **Mean (SD)** | **N (%)** |
| --- | --- | --- |
| **Age** | 52.43 (9.06) |  |
| **Sex**  Female  Male |  | 13 (92.9)  1 (7.1) |
| **Education**  Middle school  High school  University degree  Post-graduate education |  | 5 (14.3)  13 (37.1)  14 (40)  3 (8.6) |
| **Occupation**  Employed  Unemployed  Housewife  Retired |  | 9 (64.3)  2 (14.3)  2 (14.3)  1 (7.1) |
| **Pain duration**  From 1 to 3 years  From 4 to 10 years  From 10 to 20 years  More than 20 years |  | 0 (0)  2 (14.8)  6 (42.6)  6 (42.6) |

**Table 4S.** **Socio-demographic characteristics of CP specialists included in sample B (N=11).**

|  | **Mean (SD)** | **N (%)** |
| --- | --- | --- |
| **Age** | 50.64 (13.17) |  |
| **Sex**  Female  Male |  | 5 (45.5)  6 (54.5) |
| **Occupation**  Biologist  Physician  Psychologist |  | 1 (9.1)  6 (54.5)  4 (36.4) |
| **Years of experience**  5–10 years  > 10 years |  | 6 (43%)  8 (57%) |
